# MiRNA-615-5p Functions as a Tumor Suppressor in Pancreatic Ductal Adenocarcinoma by Targeting AKT2

**DOI:** 10.1371/journal.pone.0119783

**Published:** 2015-04-09

**Authors:** Yang Sun, Tingting Zhang, Cuiping Wang, Xianglan Jin, Congwei Jia, Shuangni Yu, Jie Chen

**Affiliations:** Department of Pathology, Peking Union Medical College Hospital, Chinese Academy of Medical Sciences and Peking Union Medical College, Tsinghua University, 1 Shuai Fu Yuan Hu Tong, Beijing, People’s Republic of China; Sun Yat-sen University Medical School, CHINA

## Abstract

**Background:**

Aberrant microRNA (miRNA) expression is associated with tumor development. This study aimed to elucidate the role of miR-615-5p in the development of pancreatic ductal adenocarcinoma (PDAC).

**Methods:**

Locked nucleic acid *in situ* hybridization (LNA-ISH) was performed to compare miR-615-5p expression in patients between PDAC and matched adjacent normal tissues. Effects of miR-615-5p overexpression on cell proliferation, apoptosis, colony formation, migration, and invasion were determined in the pancreatic cancer cell lines PANC-1 and MIA PaCa-2. Effects of miR-615-5p on *AKT2* were examined by dual-luciferase reporter assay. Lentivirus expressing miR-615 was used to create stable overexpression cell lines, which were subsequently used in mouse xenograft and metastasis models to assess tumor growth, apoptosis and metastasis.

**Results:**

miR-615-5p expression was significantly lower in PDAC than in adjacent normal tissues. Low levels of miR-615-5p were independently associated with poor prognosis (HR: 2.243, 95% CI: 1.190-4.227, *P*=0.013). AKT2 protein expression was inversely correlated with miR-615-5p expression (*r*=-0.3, *P*=0.003). miR-615-5p directly targeted the 3’-untranslated region of *AKT2* mRNA and repressed its expression. miR-615-5p overexpression inhibited pancreatic cancer cell proliferation, migration, and invasion *in vitro*, and tumor growth and metastasis *in vivo*. Furthermore, miR-615-5p overexpression also induced pancreatic cancer cell apoptosis both *in vitro* and *in vivo*.

**Conclusions:**

These results show that miR-615-5p inhibits pancreatic cancer cell proliferation, migration, and invasion by targeting AKT2. The data implicate miR-615-5p in the prognosis and treatment of PDAC.

## Introduction

Pancreatic cancer is the fourth leading cause of cancer deaths among men and women and is responsible for 6% of all cancer-related deaths [1]. The most common type of pancreatic cancer is pancreatic ductal adenocarcinoma (PDAC), an aggressive and highly invasive tumor type [2]. PDAC typically arises in the head of the pancreas but can infiltrate into surrounding tissues; over time, PDAC frequently metastasizes to the liver and lungs. Several risk factors are associated with PDAC development; these include advanced age, smoking, obesity, and chronic pancreatitis [3–5]. Germline mutations in a number of genes, including *INK4A*, *BRCA2*, and *MLH1* have also been shown to be responsible for inherited predisposition to the disease [6, 7]. One of the most important drivers of PDAC progression is K-RAS, which is mutated and constitutively activated in a significant percentage of tumors [8]. PDAC is often diagnosed at a late stage; thus, surgery to remove the tumor is not usually an option. Moreover, other therapies, such as radiation and chemotherapy, have been only minimally effective [9, 10]. Thus, new therapeutic strategies to treat PDAC are critical.

MicroRNAs (miRNAs) are a class of endogenously expressed, small, noncoding regulatory RNA molecules [11], which play important roles both in normal biology and in diseased states, such as cancer. Their roles in carcinogenesis are multifaceted, as they have been shown to regulate proliferation, differentiation, apoptosis, metastasis, and sensitivity to both chemotherapy and radiation [12, 13]. While some miRNAs function as oncogenes to promote tumorigenesis, others have a tumor suppressive role. As an example, miR-224 promotes proliferation and tumor growth in colorectal cancer [14]. In contrast, miR-375 is a tumor suppressor in oral cancer [15], and miR-622 functions as a tumor suppressor in lung cancer cells [16]. Importantly, a role for miRNAs in pancreatic cancer has been well established. miR-148b functions as a tumor suppressor in pancreatic cancer by targeting AMPKα1 [17].

Genome-wide miRNA expression analysis identified miR-615-5p, located within CpG islands of the HOX gene cluster on chromosome 12q13.13, as tumor-suppressor in hepatocellular carcinoma (HCC) via inhibition of IGF-II [18]. In the present study, we found that miR-615-5p expression was decreased in PDAC tissues compared to normal pancreatic tissues. We hypothesized that decreased expression of miR-615-5p may promote pancreatic cancer development and progression.

Our bioinformatics analysis found that *AKT2* contains a miR-615-5p binding site within its 3’-untranslated region (UTR). Importantly, AKT2 is a major downstream effector of phosphatidylinositol 3-kinase and considered to be a potential target for pancreatic cancer therapy [19]. *AKT2* is amplified in 10–20% of pancreatic cancers [19, 20] and activated in up to 60% of pancreatic tumors [20–22]. Active AKT2 signaling promotes cell growth and resistance to chemotherapy in pancreatic cancer [23, 24]. Thus, miRNA-mediated inhibition of AKT2 might represent a useful therapeutic strategy in *AKT2*-amplified pancreatic cancer. To begin to address this question, in this report, we explore the relationship between miR-615-5p and AKT2 in PDAC.

## Materials and Methods

### Human tissue samples and cell lines

Ninety-seven pairs of primary PDAC and adjacent normal tissues were obtained from patients between April 2008 and October 2012 in the Department of Pathology, Peking Union Medical College Hospital (China). None of the patients recruited in the present study had received radiotherapy or chemotherapy or any other treatment before operation. Clinical data and follow-up records of pancreatic cancer patients were collected in a dedicated electronic database. Patients with incomplete clinical information were excluded. The patients enrolled included 57 males and 40 females, with 47 individuals younger than 60 years and 50 participants aged 60 and above. Among them, 37 patients were of clinical stages I or IIA, whereas 60 had advanced disease (stages IIB, III and IV). Detailed information for all patients enrolled in the study is summarized in Table 1. Sample collection and all other procedures were approved by the Clinical Research Ethics Committee of Peking Union Medical College and Hospital Ethics Committee, and all participants provided written informed consent.

**Table 1:** Correlation between miR-615-5p expression and multiple clinico-pathological characteristics, including AKT2 expression, in PDAC patients.

Characteristics	miR-615-5p, N(%)			
	N	Negative	Positive	*P*
Sex				
Male	57	23 (40.4)	34 (59.6)	0.536
Female	40	19 (47.5)	21 (52.5)	
Age				
<60	47	20 (42.6)	27 (57.4)	0.886
≥60	50	22 (44.0)	28 (56.0)	
Location				
head	67	26(38.8)	41(61.2)	0.182
body/tail	30	16(53.3)	14(46.7)	
Pancreaticobiliary ductal infiltration				
Yes	44	15(34.1)	29(65.9)	0.095
No	53	27(50.9)	26(49.1)	
Peritoneal metastasis				
Yes	10	5(50)	5(50)	0.296
No	87	29(33.3)	58(66.7)	
T classification				
T1	5	3 (60.0)	2 (40.0)	0.023
T2	10	3 (30.0)	7 (70.0)	
T3	68	25 (36.8)	43 (63.2)	
T4	14	11 (78.6)	3 (21.4)	
Differentiation state				
High	19	4 (21.1)	15 (78.9)	0.047
Medium	50	26 (52.0)	24 (48.0)	
Low	28	15 (53.6)	13 (46.4)	
Clinical stage classification				
I/IIA	37	11 (29.7)	26 (70.3)	0.034
IIB/III/IV	60	31 (51.7)	29(48.3)	
Node metastasis				
N0	47	18 (38.3)	29 (61.7)	0.335
N1	50	24 (48.0)	26 (52.0)	
AKT2 expression				
Negative	49	14 (28.6)	35 (71.4)	0.003 (Kendall correlation analysis)
Positive	48	28 (58.3)	20 (41.7)	

The pancreatic cancer cell lines PANC-1 and MIA PaCa-2 were purchased from American Type Culture Collection (ATCC; Manassas, VA, USA). Cells were cultured in DMEM medium (GIBCO; San Diego, CA, USA) supplemented with 10% fetal bovine serum (GIBCO) at 37°C with 5% CO2. Every other month, all cells were tested and confirmed to be free from mycoplasma contamination. One month prior to experiments, cell lines were subjected to morphological examination, short tandem repeat (STR) analysis, growth curve analysis, and mycoplasma detection according to the ATCC cell line verification test recommendations.

### Locked nucleic acid in situ hybridization (LNA-ISH)

LNA-ISH was performed according to the manufacturer’s protocol (Exiqon, Vedbaek, Denmark). The 5’-DIG- and 3’-DIG-labeled miRCURY LNA Detection probes for human mature miR-615-5p had the sequence 5’-GATCCGAGCACCGGGGACCCCC -3’. A U6 probe was used as positive control, while a scrambled probe was used as negative control; all probes were purchased from Exiqon. Four-micrometer-thin sections of FFPE tissues were mounted onto glass slides as previously described [25] and allowed to hybridize at 61°C.

Slides were independently scored by two experienced pathologists without preliminary knowledge of clinical data. Scoring levels were negative (-), weak, focally positive (1+), and strongly positive (2+). Staining scores were determined according to the following parameters: 2+, more than 50% of tumor cells showing strong staining (similar to normal acinar cells); 1+, less than or equal to 50% of tumor cells showing staining similar to acinar cells; negative, most to all tumor cells showing a staining weaker than normal staining in acinar cells [26].

### Constructs, transfections, and dual luciferase reporter assays

We used miRecords (http://mirecords.biolead.org/) to predict potential target genes of miR-615-5p. The sequences of the predicted target sites were confirmed by comparison to TargetScanHuman (release 6.2, http://www.targetscan.org/vert_61/). To examine whether the human *AKT2* gene was a miR-615-5p target, the 3’-UTR of *AKT2* containing approximately 100 bp around the predicted microRNA binding site was amplified. PCR products were subcloned into pmirGlo Dual-Luciferase miRNA Target Expression Vectors (Promega; Carlsbad, CA, USA) downstream of Renilla luciferase using the *NotI* and *XbaI* restriction enzymes. Primers and oligonucleotides used for the Dual Luciferase Reporter Assay are provided in Table A in S1 File. The mir-615-5p miRNA mimic and a control mimic (NC) were purchased from GenePharma, Inc. (Shanghai, China).

For the Dual Luciferase Reporter Assay, MIA PaCa-2 and PANC-1 cells were co-transfected with multiple combinations of 100μg/mL pmirGlo construct and 50 nmol/L mimic. The transfection medium was replaced 6 hours post-transfection. Twenty-four hours after transfection, firefly and Renilla luciferase activities were measured using the Dual-Luciferase Reporter Assay (Promega, USA).

### Western blotting

For Western blotting, MIA PaCa-2 and PANC-1 cells were transfected with mir-615-5p mimic or control mimic (50 nmol/L) using the Lipofectamine 2000 reagent (Invitrogen). The transfection medium was replaced 6 hours post-transfection. Seventy-two hours after transfection, cells were lysed in Radio-Immunoprecipitation Assay (RIPA) buffer (Pierce Biotechnology, IL, USA), and protein concentrations in the lysates were determined by the BCA method. Sixty micrograms of total protein were separated on 12% SDS–PAGE gels and transferred to PVDF membranes (0.45um; Bio-Rad, CA, USA). After blocking with 5% milk powder diluted in TBS containing 0.05% Tween 20 (TBST), membranes were blotted with primary antibodies against AKT2 (AM1848b; Abgent, SD, USA) and β-actin (SC-47778, Santa Cruz Biotech; USA). Horseradish peroxidase (HRP)-conjugated secondary antibody (Santa Cruz Biotech; USA) was used for detection using an enhanced chemiluminescence (ECL) system (Amersham Pharmacia Biotech, Bucks, UK).

### Cell proliferation and apoptosis assays

Cell proliferation was monitored using the Cell Counting Kit-8 (CCK-8) (Dojindo Laboratories, Kumamoto, Japan), at 24, 48, 72, 96, and 120 hours after Lipofectamine 2000-mediated transfection with either mir-615-5p mimic or control mimic (50 nmol/L). Two hours after cells were incubated in 10% (v/v) CCK-8 (diluted in culture medium) at 37°C, the spectrophotometric absorbance of each sample was measured at both 450 nm (test wavelength) and 630 nm (reference wavelength), according to the manufacturer’s instructions. Absorbance was read using a Vmax microplate spectrophotometer (Molecular Devices, Sunnyvale, CA).

The apoptosis assay was performed in both MIA PaCa-2 and PANC-1 cell lines 48 hours after transfection of mir-615-5p mimic or control mimic (50 nmol/L) using Lipofectamine 2000. Samples were processed using the Annexin V-FITC Apoptosis Detection Kit I (BD Biosciences, NJ, USA) and analyzed on an Accuri C6 Flow Cytometer.

Forty-eight hours after transient transfection with mir-615-5p mimic or control mimic (50 nmol/L), Panc-1 and MIA PaCa-2 cells were labeled with the 5-ethynyl-2’-deoxyuridine (EdU) reagent (50μM)[27]. Labeling was performed for 2 hours using the Cell Light EdU DNA imaging Kit (Ruibo Biotech, Guangzhou, China). Afterward, cell nuclei were stained with Vectashield DAPI (4’6-diamidino-2-phenylindole 2HCl; Vector Labs, USA) mounting media. Images were acquired and analyzed with a fluorescent microscope (AMG EVOS; FL, USA). The percentage of EdU-positive cells was calculated according to the following equation: (EdU-labeled cells/DAPI stained cells) × 100%.

### Soft-agar colony formation, cell migration, and invasion assays

A 1.5-mL base layer of agar (0.5% agar in DMEM with 10% FBS) was allowed to solidify in a six-well flat-bottom plate before the addition of a 1.5 mL cell suspension containing 4,000 cells in 0.35% agar in DMEM with 10% FBS. The cell-containing layer was then solidified at 4°C for 20 minutes. Colonies were allowed to grow for 21 days at 37°C with 5% CO_2_ before imaging.

A wound-healing assay was performed to assess effects on cell migration. An artificial wound was created on a confluent cell monolayer using a 200μL pipette tip 24 hours after transient transfection. Mitomycin C was added to the culture wells (final concentration for PANC-1, 10 μg/mL; for MIA PaCa-2, 20 μg/mL) to prevent proliferation. Images of the migration/wound healing process were captured at 0, 12, 24, 36, 48, and 60 hours [28].

Invasion was evaluated by the ability of cells to pass through a Matrigel-coated membrane matrix (BD Biosciences, NJ, USA). Twenty-four hours after transient transfection, cells were seeded onto a Matrigel-coated membrane matrix in a 24-well culture plate. Fetal bovine serum was added to the lower chamber as a chemoattractant. After 24 hours, non-invading cells were removed. Invasive cells, located on the lower surface of the chamber, were stained with 0.1% crystal violet (Sigma—Aldrich, MO, USA) and counted.

### Lentivirus packaging and transduction

The miR-615 sequence (nucleotides 54427734–54427829 of chromosome 12; GenBank Accession number NC_000012.11) was PCR amplified from genomic DNA of adjacent normal tissues and cloned into the *EcoRI* and *XcmI* sites downstream of GFP in the lentiviral expression vector FUGW (Cal Tech, CA, USA) (pLV-miR-615) [29, 30]. Primer sequences for miR-615 were as follows: 5'- CTGTGTGCGCCCAAATTTACGACG-3’ (forward) and 5'-CGGAATTCAGGGGTGAATAGCTTGCAGCGTTCC-3' (reverse). pLV- miR-615 or pLV-miR-mock were co-transfected with pLP1, pLP2, and pLP/VSVG (Viral Packaging Kit, Invitrogen) using the Fugene HD lenti transfection reagent (Roche, CHE), according to the manufacturer’s instructions. Virus packaging was performed in 293T cells. Forty-eight hours after transfection, the supernatant was harvested and cleared by centrifugation at 500×*g* for 10 min. The MIA PaCa-2 cells were transduced with the lentivirus containing pLV-miR-615 or pLV-miR-mock. Forty-eight hours after infection, 2 μg/ml of puromycin was added to the medium for 2 weeks to select for cells that were successfully infected with the lentivirus. The selection was performed based on FITC fluorescence using a flow cytometer (Accuri C6 Flow Cytometer; BD Accuri Cytometers, NJ, USA). The cell line stably overexpressing miR-615 was denoted LV-miR-615-MIA, and the mock vector cell line named LV-control-MIA.

TaqMan microRNA qRT-PCR assays were used to measure the expression of miR-615-5p in the two stable cell lines using GeneAmp PCR System 9700 (Applied Biosystems, CA, USA). TaqMan MicroRNA Reverse Transcription Kit, TaqMan MicroRNA Assays (ID: 002353), and TaqMan Gene Expression Master Mix were purchased from Applied Biosystems (CA, USA).

### Pancreatic tumor xenograft and metastasis models

Six-week-old male BALB/c nude mice (body weight: 20.34±2.19) (n = 32) were purchased from Vital River Laboratory Animal Technology Co., Ltd., (Beijing, China), and used to examine tumorigenicity and metastasis.

LV-miR-615-MIA or LV-control-MIA cells (6 × 10^6^ cells in 200ul PBS per mouse) were subcutaneously inoculated into the dorsal flanks of mice (10 LV-control-MIA cells and 10 stably overexpressing mir-615-MIA cells). Tumor dimensions were measured by calipers every 3 days, and the volumes estimated using the formula for hemi-ellipsoids: V = length (mm) × width (mm) × height (mm) × 0.5236 [25]. For end-point experiments, tumors were removed and weighed 6 weeks after tumor cell injection. Two independent reviewers were blinded to the grouping when measuring tumor volumes and weights.

To further confirm the effect of mir-615 on the metastatic potential of MIA cells, LV-miR-615-MIA or LV-control-MIA cells (1 × 10^5^ cells resuspended in 200 μL PBS per mouse) were injected by tail-vein to nude mice (6 LV-control-MIA cells and 6 stably overexpressing mir-615 MIA cells) after intraperitoneal injection of 3% pentobarbital sodium (2 ml/kg body weight) for anesthesia [31]. Four weeks after injection, animals were euthanized by cervical dislocation, and livers were harvested to count tumor nodules and evaluate tumor metastasis. Two independent pathologists were blinded to the grouping when counting tumor nodules in the liver tissues. All procedures and animal experiments were approved by the Animal Care and Use Committee of Peking Union Medical College (Approval No. XHDW-2012-016) and conducted in accordance with all state regulations.

### Immunohistochemistry, immunofluorescence, and TUNEL assays

Immunohistochemical staining of AKT2 in PDAC sections was performed using the murine monoclonal antibody against human AKT2 (1:100, AM1848b; Upstate; Abgent, USA) as previously described [32]. The AKT2-specific immunoreactivity was independently assessed as “diffusely positive”, “focally positive,” or “negative” by two experienced pathologists without preliminary knowledge of clinical data.

Indirect immunofluorescence staining of Ki67 was used on mouse tumor tissue sections [33]. Deparaffinization, endogenous peroxidase inactivation, antigen retrieval of FFPE clinical tissues, and immunostaining with mouse monoclonal antibodies (sc-23900, Santa Cruz Biotech, USA) were performed. Goat anti-mouse IgG secondary antibodies conjugated to rhodamine (115-025-003, Jackson, USA) were used for detection.

Apoptosis in mouse tumor tissues was measured using the In Situ Cell Death Detection Kit (1684809, Roche, CHE), according to the supplier’s instructions.

All sections were independently evaluated by two pathologists in conjunction with the H&E-stained sections from the same lesions.

### Statistical analyses

Statistical analyses were performed using the SPSS statistical software 17.0 (SPSS Inc., Chicago, IL, USA). Normally distributed data are presented as mean ± standard deviation (SD) of at least three independent experiments. Statistical significance was evaluated by one-way analysis of variance (ANOVA) with Student-Newman-Keuls (SNK) test for post hoc test or Student's *t* test. Categorical variables were compared using Fisher’s exact test. Kendall correlation analysis was used for correlation between miR-615-5p and AKT2 expression. The Kaplan-Meier and log-rank tests were used for the cumulative survival analysis. The Cox regression analysis was used for multivariate survival analysis. A two-tailed *P*-value of less than 0.05 was considered statistically significant (*P* < 0.05).

## Results

### 1. miR-615-5p expression and its association with clinico-pathological characteristics in PDAC patients

**Fig 1:**
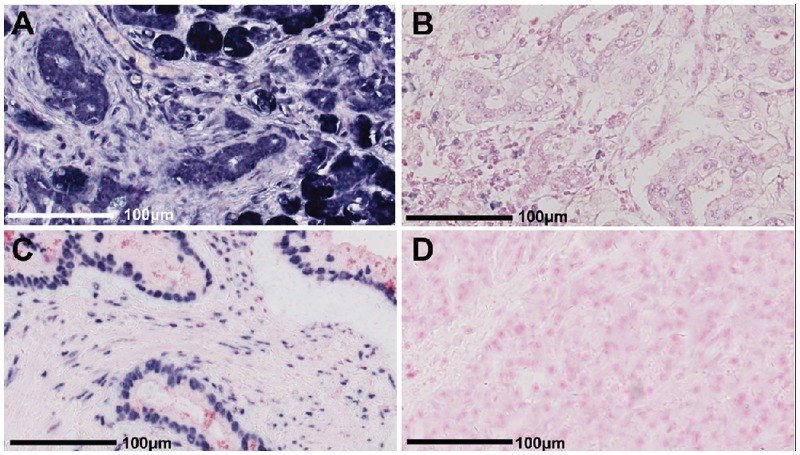
Locked nucleic acid *in situ* hybridization (LNA-ISH) shows differential expression of miR-615-5p in pancreatic ductal adenocarcinoma (PDAC) compared to normal pancreatic tissues. Bar = 100 μm. (A) Strong positive (2+) staining of miR-615-5p in normal pancreas; note the dark acinar nuclear staining with cytoplasmic stippling. (B) Weak positive (+) staining of miR-615-5p in PDAC tissue. (C) Positive control staining for the U6 probe in PDAC tissue; note the dark nuclear staining observed in cancer cells. (D) Negative control for the scrambled probe in normal pancreatic tissue; no positive staining is observed.

We performed LNA-ISH to examine the expression of miR-615-5p in PDAC samples and adjacent normal pancreatic tissues. The U6 probe was used as a positive control, while the scrambled probe served as a negative control (Fig 1C and Fig 1D). MiR-615-5p was primarily expressed in acinar cells. There was strong positive (2+) expression of miR-615-5p in normal pancreatic tissues; note the dark acinar nuclear staining with cytoplasmic stippling (Fig 1A). Weak positive (+) expression of miR-615-5p was detected in PDAC tissues (Fig 1B). Staining of miR-615-5p by LNA-ISH was manually scored by cytoplasmic intensity. As shown in Table 2, miR-615-5p was positively expressed in 90 cases of normal pancreatic ductal epithelium (90/97, 92.8%). In contrast, miR-615-5p was only expressed in 52 cases of PDAC cells (52/97, 53.6%) (*P*<0.01).

**Table 2:** miR-615-5p expression in PDAC and normal pancreas tissues determined by locked nucleic acid *in situ* hybridization (LNA-ISH).

miR-615-5p expression	PDAC (n = 97)	Normal pancreas (n = 97)
Negative	45	7
Weak positive	29	40
Strong positive	23	50
Positive rate	53.6%(52/97)	92.8%(90/97)**

***Note*:** **P <0.01 PDAC tissue *vs*. normal pancreas tissues (Fisher’s exact test). PDAC: Pancreatic ductal adenocarcinoma.

Subsequently, we analyzed the correlation between positive miR-615-5p expression and various clinico-pathologic characteristics that may affect the prognosis of PDAC patients. We found that miR-615-5p expression was correlated with T classification, differentiation and clinical stage classification (all *P*<0.05). Age, sex, and other clinical variables, however, were not correlated with miR-615-5p expression in PDAC patients (*P*>0.05) (Table 1).

### 2. High MiR-615-5p expression is associated with better cumulative survival

**Fig 2:**
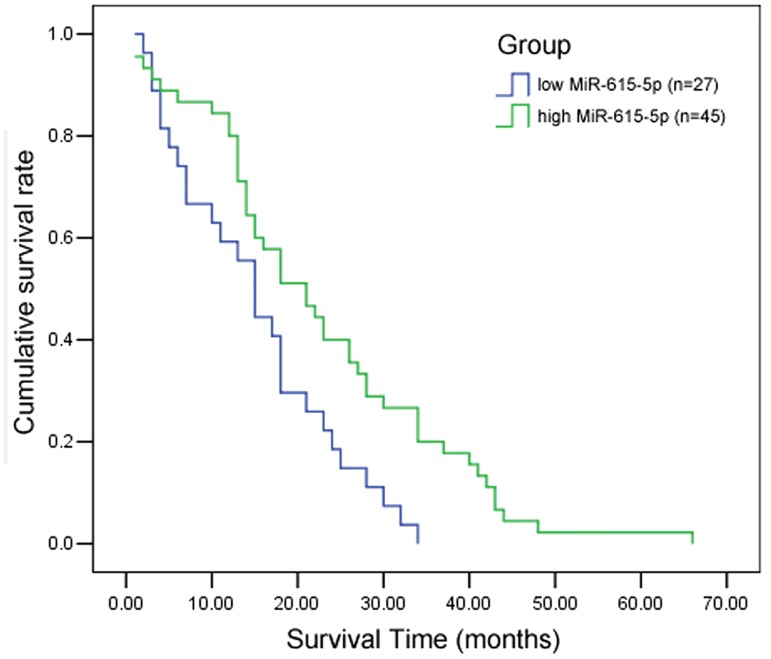
Kaplan-Meier analysis demonstrates that PDAC patients with low-miR-615-5p expression have a lower overall survival than those with high expression of miR-615-5p (log-rank test, *P*<0.01).

Sixty-six month follow-up data were available for 72 PDAC patients (Table B in S1 File), of which 45 (45/72, 62.5%) were miR-615-5p positive; of these, 17 (17/45, 37.8%) survived to the end of the follow-up period. Therefore, the survival time of miR-615-5p positive patients was 22.8 ± 14.4 months. There were 27 (27/72, 37.5%) miR-615-5p negative patients. Of these, 6 (6/27, 22.2%) survived to the end of the follow-up period, representing a survival time of 15.1 ± 9.6 months. Univariate analysis using the Kaplan-Meier method indicated that the cumulative survival rate of PDAC patients with high miR-615-5p expression (n = 45) was significantly higher than that of patients with low miR-615-5p levels (n = 27) (log-rank test: *P*<0.01) (Fig 2).

Multivariate Cox regression analysis was conducted to determine the independent prognostic effects of the various clinico-pathologic parameters. Our results demonstrated that location of PDAC within the head region, pancreaticobiliary ductal infiltration, low expression of miR-615-5p, and clinical stages IIB/III/IV were independent factors for poor prognosis of PDAC (all *P*<0.05, Table 3).

**Table 3:** Multivariate Cox regression analysis of clinico-pathological variables and cumulative survival of patients with PDAC.

variables	HR	95% CI	*P*
Gender (male *vs*. female)	1.407	0.746–2.654	0.291
Age (years) (≥60 *vs*.<60)	0.747	0.392–1.424	0.375
Location (head *vs*. body/tail)	5.058	2.012–12.717	0.001
Pancreaticobiliary ductal infiltration	3.599	1.752–7.391	0.000
Peritoneal metastasis	1.369	0.462–4.060	0.571
miR-615-5p(low *vs*. high)	2.243	1.190–4.227	0.013
Clinical stage classification (IIB/III/IV *vs*. I/IIA)	0.493	0.244–0.996	0.049

***Note***: HR: hazard ratio; 95% CI: 95% confidence interval.

### 3. miR-615 overexpression inhibits tumor growth and metastasis *in vivo*

**Fig 3:**
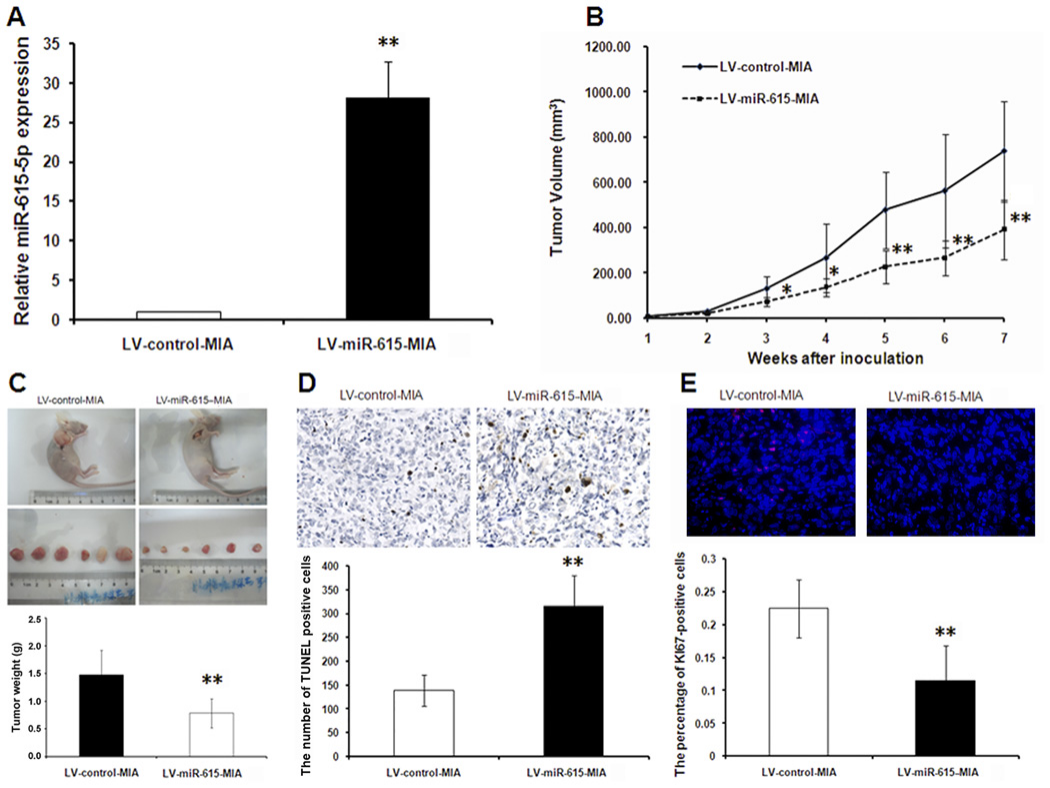
MiR-615 overexpression inhibits tumor growth *in vivo*. MIA PaCa-2 cells transduced with lentivirus pLV-miR-615 (LV-miR-615-MIA) or pLV-miR-mock (LV-control-MIA) (6 × 10^6^ cells) were subcutaneously inoculated into the dorsal flanks of BALB/c nude mice. (A) Relative miR-615-5p expression was determined by TaqMan microRNA qRT-PCR assay. (B) Tumor volume (cm^3^) was assessed by calipers every week. (C) Tumor weights (g) measured at the end of the experiment were significantly reduced in the LV-miR-615-5p-MIA group. (D) The extent of apoptosis was determined in tumor tissue by TUNEL staining (scale bar: 600um). (E) Determination of Ki-67 in tumor tissues by immunofluorescence (Magnification:×100). Red: Rhodamine; Blue: DAPI. The numbers of apoptotic cells (D) and the percentages of Ki-67-positive cells (E) were determined from 10 randomly selected fields in a single sample. Two independent reviewers were blinded to the grouping when measuring the numbers of apoptotic cells and the percentages of Ki-67-positive cells. The data are shown as mean±standard deviation (SD) (n = 8 in LV-control-MIA group; n = 6 in LV-miR-615-MIA group). *P<0.05, **P<0.01 *vs*. LV-control-MIA group.

TaqMan microRNA qRT-PCR assays were used to measure the expression of miR-615-5p in the two stable cell lines. As shown in Fig 3A, miR-615-5p expression was significantly elevated in the LV-miR-615-MIA group compared with the LV-control-MIA group. MIA PaCa-2 cells stably overexpressing miR-615 (LV-miR-615-MIA) or control cells (LV-control-MIA) were injected into the dorsal flanks of nude mice. Three weeks after inoculation, the tumor formation rate in the LV-miR-615-5p-MIA group was lower than in control animals (58.3 *vs*. 88.3%). At the 7-week time point, tumor volume (393.10±132.20 *vs*. 738.04±222.12 mm^3^, *P*<0.01) and tumor weight (0.79±0.26 vs. 1.48±0.44 g, *P*<0.01) were significantly decreased in mice overexpressing miR-615 compared with the controls (Fig 3B and Fig 3C). In agreement, an increased number of apoptotic cells (determined by TUNEL analysis) (Fig 3D) and a decreased percentage of Ki-67-positive cells (determined by immunofluorescence staining) (Fig 3E) were observed in tumor tissues from the miR-615 overexpression mice compared with controls. Thus, miR-615-5p overexpression may inhibit pancreatic cancer growth by blocking proliferation and promoting apoptosis.

**Fig 4:**
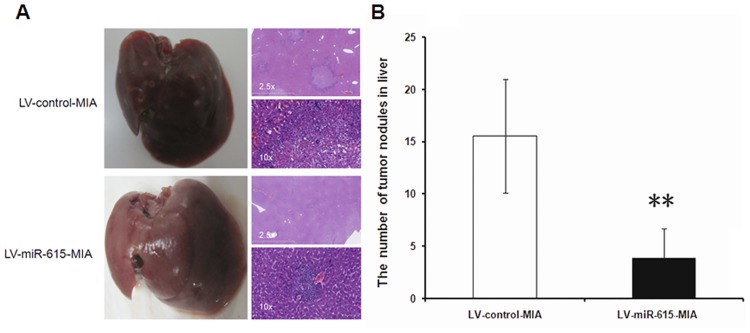
MiR-615-5p overexpression inhibits tumor metastasis *in vivo*. LV-miR-615-5p-MIA or LV-control-MIA cells were injected into nude mice via tail vein. Mouse livers were harvested to evaluate tumor metastasis 4 weeks after injection. (A) Mouse liver tissue (left panel) and representative tumor nodules (right panel, H&E staining) (Magnification: ×2.5, upper; ×10, lower). (B) The number of tumor nodules in the liver was quantified and is presented as mean±SD (n = 6 in LV-control-MIA group; n = 6 in LV-miR-615-5p-MIA group). **P<0.01 *vs*. LV-control-MIA group.

When LV-miR-615-MIA and LV-control-MIA cells were injected into nude mice by tail vein, the LV-miR-615-MIA animal group showed a reduced number of tumor nodules in the liver compared with mice receiving control cells (3.8±2.8 *vs*. 15.5±5.4, *P*<0.01) (Fig 4A and Fig 4B). These data support a role for mir-615-5p in suppressing tumor metastasis.

### 4. miR-615-5p overexpression inhibits pancreatic cancer cell proliferation and induces apoptosis *in vitro*

**Fig 5:**
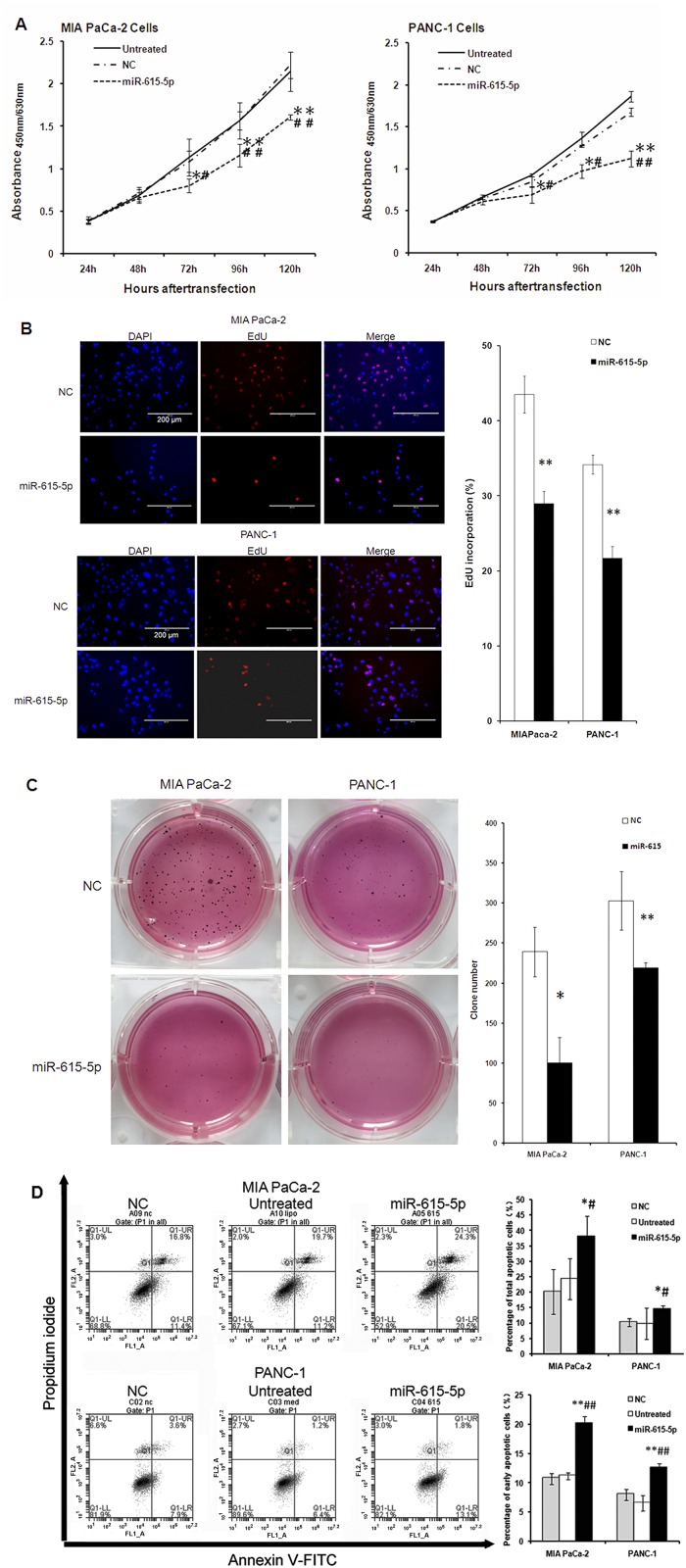
Effects of miR-615-5p overexpression on cell proliferation, colony formation, and apoptosis in pancreatic PANC-1 and MIA PaCa-2 cancer cell lines. PANC-1 and MIA PaCa-2 cells were transiently transfected with the miR-615-5p miRNA mimic (miR-615-5p) or a control construct (NC). (A) Cell proliferation was measured by CCK-8 detection at 24, 48, 72, 96, and 120 hours after transfection both in MIA PaCa-2 and PANC-1 cells. (B) Cell proliferation was also determined by 5-ethynyl-2’-deoxyuridine (EdU) staining 48 hours after transfection (Scale bar: 200 μm). Red: EdU; Blue: DAPI. (C) Colony formation assay after 21 days in culture. (D) Cell apoptosis was determined by flow cytometry analysis using Annexin V/PI staining 48 hours after transfection. The data are shown as mean±SD. *P<0.05 *vs*. untreated cells or NC group.

To examine the role of miR-615-5p in pancreatic cancer cell proliferation, both MIA PaCa-2 and PANC-1 cells were transfected with miR-615-5p mimic or a control mimic (NC). CCK-8 assay data showed that growth was significantly inhibited in both cell lines transfected with miR-615-5p compared with either the control transfected group or untreated cells (Fig 5A). Consistent with these findings, results from EdU assay showed miR-615-5p overexpression significantly inhibited pancreatic cancer cell proliferation (EdU incorporation: 21.67±1.56 *vs*. 34.18±1.28%, *P*<0.01 in PANC-1; 28.95±1.65 *vs*. 43.50±2.46%, *P* = 0.01 in MIA PaCa-2) (Fig 5B). Furthermore, both MIA PaCa-2 and PANC-1 cells transfected with miR-615-5p mimic displayed fewer and smaller colonies compared with the NC groups in an anchorage-independent growth assay (both *P*<0.05, Fig 5C).

In MIA PaCa-2 cells transfected with a control mimic, the percentages of early and total apoptotic cells were 10.7±1.0 and 20.2±7.3%, respectively; these percentages increased to 20.0±1.3 and 38.0±6.6%, respectively, in cells transfected with miR-615-5p mimic. Similar findings were obtained with the PANC-1 cell line. Control PANC-1 cells displayed early and total apoptotic percentages of 8.0±1.0 and 10.2±1.2%, respectively, which increased to 12.5±0.8 and 14.5±1.2%, respectively following transfection with the miR-615-5p mimic (Fig 5D).

### 5. Overexpression of miR-615-5p inhibits pancreatic cancer cell migration and invasion in *vitro*

**Fig 6:**
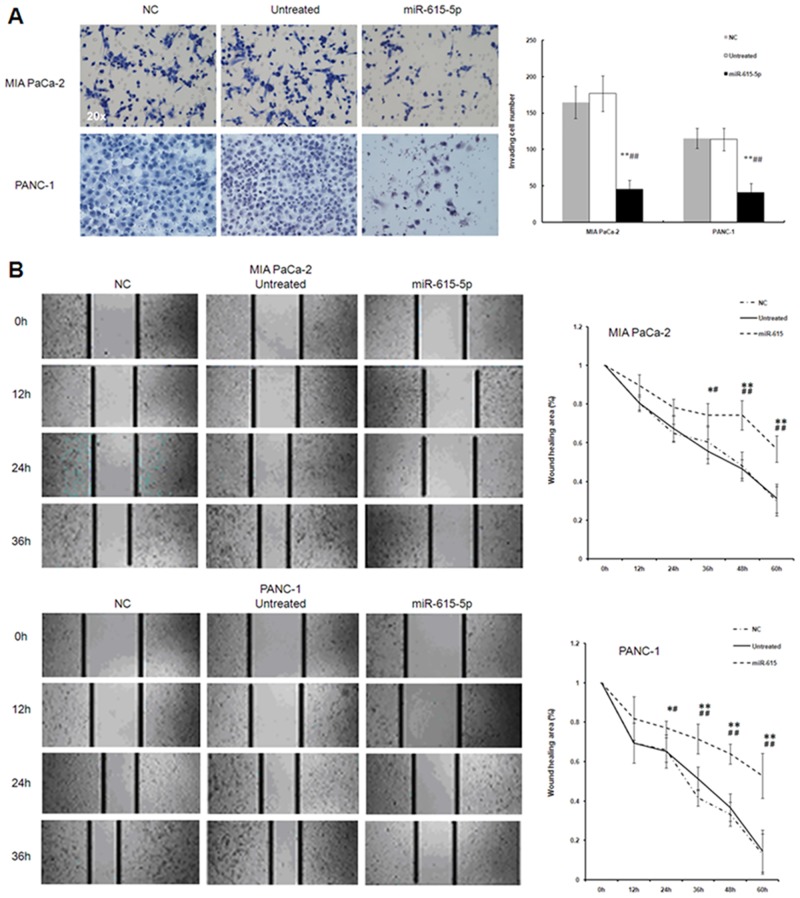
Effects of miR-615-5p overexpression on cell invasion and migration in pancreatic PANC-1 and MIA PaCa-2 cancer cell lines. PANC-1 and MIA PaCa-2 cells were transiently transfected with the miR-615-5p miRNA mimic (miR-615-5p) or a control mimic (NC). (A) Cell invasion was determined by transwell chamber assay 48 hours after transfection; cells that successfully invaded through the chamber were stained with 0.1% crystal violet and counted. Staining is shown on the left (Magnification: ×100) and quantification on the right; (B) Cell migration was assessed by performing a wound-healing assay at 0, 12, 24, 36, 48, and 60h after transfection. Results are presented as rate of the wound area filled at a given time. The data are shown as mean±SD. *P<0.05 *vs*. untreated PANC-1 and MIA PaCa-2 cells, respectively; #*P*<0.05 *vs*. NC group. Representative images are shown on the left (Magnification: ×100), and quantification is provided on the right.

The effects of miR-615-5p on migration and invasion of MIA PaCa-2 and PANC-1 cells were assessed. As shown in Fig 6A, results from a transwell invasion assay showed that overexpression of miR-615-5p significantly reduced invading MIA PaCa-2 and PANC-1 cell numbers compared with the NC group (64.1% reduction in PANC-1 cells; 72.2% reduction in MIA PaCa-2 cells). As far as morphology is concerned, untreated MIA PaCa2 cells were scattered, well-adherent with clear cell outline, spindle or fusiform shaped, plump with cytoplasm. After transfection with miR-615-5p, cells shrank, were round with increased intracellular particles, and were poorly refractive with some cells floating in the medium (data not shown). Untreated PANC-1 cells were mostly polygonal; some cells were spindle, diamond or teardrop-shaped; they presented flaky arrangement, clear cell outline, and adherent growth. After transfection with miR-615-5p, intracellular particles increased; the cells became sleek with reduced cytoplasm, the cell gap widened and translucent cells appeared between small particles (data not shown).

We next performed a wound healing assay to determine the effect of miR-615-5p on cell migration; the assay was performed in the presence of mitomycin C to block cell proliferation. Sixty hours after transfection, we found that wound areas in MIA PaCa-2 and PANC-1 cells transfected with miR-615-5p mimic (56.9±6.8 and 52.8±11.4%, respectively) were significantly larger than those in cells transfected with a control mimic (29.9±7.6 and 13.2±10.1%, respectively) (Fig 6B).

### 6. miR-615-5p is a negative regulator of AKT2 in PDAC

**Fig 7:**
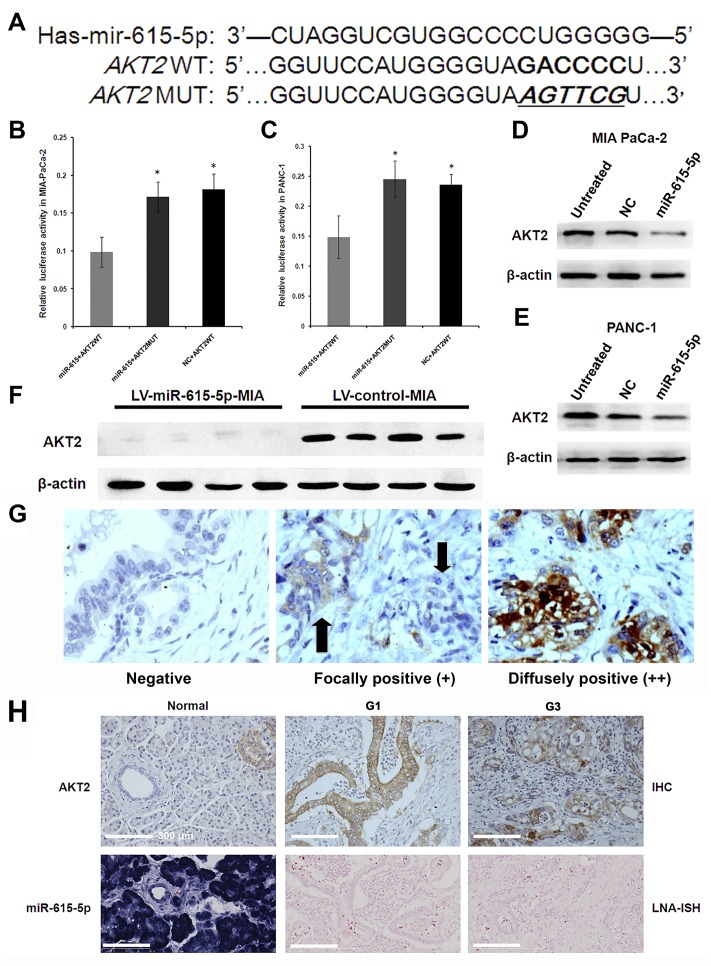
AKT2 is a direct target of miR-615-5p. (A) Schematic representation of the firefly luciferase reporter construct containing the 3’-UTR of AKT2 with either wild-type (WT) or mutant (MUT) miR-615-5p target site. The mutant construct contains mutations in 6 nucleotides within the seed region of the target site to disrupt binding of miR-615-5p. PANC-1 (B) and MIA PaCa-2 (C) cells (0.5 × 10^6^ cells per well) were transiently co-transfected with reporter plasmids (200 ng, WT or MUT) and 100 nM of either miR-615-5p miRNA mimic (miR-615-5p) or control mimic (NC) for 24 h. Protein lysates were then prepared and relative luciferase activity was determined using the dual-luciferase assay system. Renilla luciferase was used for normalization of transfection efficiency. Data are represented as normalized fold change of luciferase activity. The data are shown as mean±SD. *P<0.05 *vs*. miR-615+AKT2WT. AKT2 protein expression in MIA PaCa-2 (D) and PANC-1 (E) cells transiently transfected with mir-615-5p miRNA mimic (miR-615-5p) or control mimic (NC) were determined by Western blot. (F) AKT2 protein expression in tumor tissues from nude mice stably overexpressing miR-615-5p was determined by Western blot. β-actin was used as a loading control. (G) Higher magnification after AKT2 detection in PDAC tissues by immunohistochemistry (IHC) (Magnification: ×200); negative, focally positive and diffusely positive cells can be observed. Two arrows point to two group of cancer cells, one with positive AKT2 expression (left) and the other AKT2 negative (right). (H) AKT2 expression is inversely correlated with miR-615-5p in PDAC tissues. AKT2 expression was determined by IHC (upper panel). miR-615-5p expression was determined by locked nucleic acid *in situ* hybridization (LNA-ISH) (lower panel). Magnification: ×200. Normal: normal pancreas; PC G1: well differentiated PDAC tissue; PC G3: poorly differentiated PDAC tissue. LNA-ISH and immunohistochemistry slides were counterstained with nuclear fast red and hematoxylin, respectively.

We examined miRecords [34] and found a large number of potential miR-615-5p target genes. Among these candidates, AKT2 was selected for further analysis. We first constructed reporter plasmids harboring either the wild-type 3’-UTR region of AKT2 or a mutant 3’-UTR predicted to be insensitive to miR-615-5p (Fig 7A). PANC-1 and MIA PaCa-2 cells were co-transfected with reporter plasmids and either miR-615-5p mimic or a control mimic (NC). miR-615-5p overexpression reduced luciferase expression from the reporter plasmid containing the wild-type AKT2 3’-UTR sequence (39.4% reduction in PANC-1 cells; 42.6% reduction in MIA PaCa-2 cells) (both *P*<0.01 *vs*. NC+AKT2 MUT) (Fig 7B and Fig 7C). In contrast, as predicted, the mutated 3’-UTR of AKT2 was insensitive to miR-615-5p-mediated repression (Fig 7B and Fig 7C). Taken together, these results demonstrated that AKT2 is a target gene of miR-615-5p, and the conserved region within the 3’-UTR of AKT2 contains a functional binding site for miR-615-5p in PDAC cells.

These reporter assays were substantiated with Western blot data from MIA PaCa-2 and PANC-1 cells transfected with miR-615-5p mimic and a control mimic (NC). miR-615-5p overexpression dramatically decreased AKT2 protein levels (Fig 7D and Fig 7E). Tumor tissues from nude mice of the LV-miR-615-5p-MIA group showed a significant decrease in AKT2 protein expression as well, compared with the LV-control-MIA group (Fig 7F).

To further determine if there was any correlation between expression of AKT2 and miR-615-5p in PDAC patients, we performed immunohistochemical staining for AKT2 in 97 pairs of primary pancreatic tumor tissues and adjacent normal tissues. Higher magnification images of “diffusely” and “focally” AKT2-positive cells in PDAC tissues are shown in Fig 7G. AKT2 protein expression was elevated in tumor tissues compared with adjacent normal tissues, and further increased with the degree of tumor differentiation. In contrast, miR-615-5p showed decreased expression in tumor tissues compared with adjacent normal tissues (Fig 7H). Furthermore, AKT2 expression was inversely correlated with miR-615-5p expression (*r* = -0.3, *P* = 0.003) (Table 1).

## Discussion

In the present study, we demonstrated that miR-615-5p expression is decreased in PDAC tissues compared with adjacent normal pancreatic tissues. We also discovered that AKT2 is negatively regulated by miR-615-5p. Thus, the data provided here support a mechanism by which miR-615-5p suppresses tumorigenesis in PDAC patients.

miRNA expression profiles are distinct among different tumor types and often reflect the tissue of origin and differentiation state of the tumor [35, 36] Previous reports have shown that miR-615-5p is overexpressed in prostate cancer [37]. In contrast, this miRNA is down-regulated in PDAC [38] and HCC [18]. In the present study, we confirmed that miR-615-5p expression is decreased in clinical PDAC samples compared with normal pancreatic tissues. Additionally, the LNA-ISH method allowed us to distinguish between miR-615-5p expression in tumor cells and acinar cells [39, 40]. We also found that miR-615-5p expression was correlated with T classification, differentiation, and clinical disease stage (all *P*<0.05). Finally, while assessing follow-up information available for 72 PDAC patients, we importantly found that patients with high expression of miR-615-5p had significantly longer survival time than those with low expression. Taken together, our data support a tumor suppressive role for miR-615-5p in PDAC. Interestingly, miR-615 is located in a CpG island within the HOX gene cluster, which has been shown to be critical for both normal pancreatic development and pancreatic cancer [41]. The HOXC cluster has been previously shown to be silenced via long-range hypermethylation [42]. Recently, it was demonstrated that abnormal miR-615-5p downregulation due to promoter hypermethylation limits its inhibition of IGF2 and other targets in PDAC cells, resulting in increased tumor growth, invasion and migration capabilities [43].

To support our clinical findings, we performed several *in vivo* mouse xenograft experiments. When mice were inoculated with cell lines stably overexpressing miR-615, they displayed reduced tumor growth, decreased tumor volume, decreased proliferation, and increased apoptosis compared with animals inoculated with control cells. Moreover, fewer tumor nodules were found in the livers of mice injected with miR-615 overexpressing cells, suggesting that the microRNA suppresses tumor metastasis. Taken together, the mouse xenograft data were consistent with our clinical findings and support a role for miR-615-5p as a tumor suppressor in PDAC. miR-615-5p attenuates the malignant behavior of PDAC cells, thus, it may be a promising therapeutic target in the future. However, it should be noted that the experimental metastasis model described here does not recapitulate the metastasis process from an orthotopic primary tumor, neither does the subcutaneous injection mimic perfectly the early events of PDAC clinical tumor development. Thus, orthotopic injection of tumor cells will be used in our future work to better mimic what is seen clinically in PDCA.

While *in vivo* studies examining a role for miR-615-5p in the development of PDAC are extremely limited, a recent study from Gao et al. [43] supports the data presented here. Furthermore, a study by El Tayebi et al. [18] showed that overexpression of miR-615-5p in HCC cell lines significantly decreases cell growth and migration. Consistent with this, in our study, we observed similar effects in pancreatic cancer cells. Overexpression of miR-615-5p reduces PDAC cell proliferation, invasion, and migration, and promotes apoptosis *in vitro*.

miRNAs regulate the expression of many protein-coding genes by targeting their mRNAs for cleavage or translational repression [44]. We used miRecords to find potential targets of miR-615-5p and identified AKT2 as potential candidate. AKT2 is an important regulator of pancreatic cancer development [19] and, thus, an interesting link between miR-615-5p and PDAC prognosis. Following overexpression of miR-615-5p in PANC-1 and MIAPaCa-2 cells, and in nude mouse xenograft and metastasis models, AKT2 expression was significantly decreased. Furthermore, miR-615-5p and AKT2 were inversely correlated in human clinical PDAC samples, suggesting that loss of miR-615-5p and subsequent upregulation of AKT2 may be an important driver of PDAC progression. Finally, dual-luciferase reporter gene assays also provided evidence that AKT2 is a direct target of miR-615-5p. Taken together, these findings demonstrate that miR-615-5p regulates AKT2 expression and functions as a tumor suppressor in PDAC development.

Pancreatic cancer is a highly malignant disease and a major cause of cancer-related death [45, 46]. The best, most effective biomarkers are stable in patient samples and closely associated with clinical outcomes, particularly patient survival. miRNAs are relatively stable compared to other biological macromolecules, as they can be well preserved in tissue samples even after formalin-fixation and paraffin-embedding [47]. A number of miRNAs have demonstrated valuable prognostic prediction power in cancer patients. For example, overexpression of miR-196a predicts poor outcome in pancreatic carcinoma [48]; miR-210 is an independent prognostic factor for breast cancer and inversely correlated with disease-free and overall survivals [49]. Since a single miRNA can regulate multiple genes [44], its expression may more precisely and effectively predict disease outcome. The results from our study indicate that the expression of miR-615-5p is associated with clinical stage, differentiation status, tumor stage, and overall survival in PDAC patients.

A molecular understanding of PDAC tumorigenesis is crucial for the development of improved therapeutic strategies. Here, we identify miR-615-5p as a tumor suppressor in PDAC. The inhibitory properties of this miRNA are, at least in part, due to suppression of AKT2. Overall, these results provide novel insights into PDAC biogenesis, with implications for prediction of clinical outcome.

## Supporting Information

S1 Checklist ARRIVE: Animal research: Reporting in vivo experiments. (DOC)

S1 File Contains, Table A: Primers and oligonucleotides used for the Dual Luciferase Reporter Assay. Table B: Correlation between miR-615-5p expression and multiple clinicopathological characteristics, including AKT2 expression, in 72 follow-up PDAC patients.
(DOCX)

## References

[1] National Comprehensive Cancer Network. NCCN Clinical Practice Guidelines in Oncology. Pancreatic Adenocarcinoma, v.1. Available: http://www.nccn.org/professionals/physician_gls/pdf/pancreatic.pdf. Accessed 2015 Feb 3.

[2] Stathis A and Moore MJ. Advanced pancreatic carcinoma: current treatment and future challenges. Nat Rev Clin Oncol. 2010;7: 163–172. 10.1038/nrclinonc.2009.236 20101258

[3] Fuchs CS, Colditz GA, Stampfer MJ, Giovannucci EL, Hunter DJ, Rimm EB, et al A prospective study of cigarette smoking and the risk of pancreatic cancer. Arch Intern Med. 1996;156: 2255–2260. 8885826

[4] Gapstur SM, Gann PH, Lowe W, Liu K, Colangelo L and Dyer A. Abnormal glucose metabolism and pancreatic cancer mortality. JAMA. 2000;283: 2552–2558. 10815119 10.1001/jama.283.19.2552

[5] Michaud DS, Giovannucci E, Willett WC, Colditz GA, Stampfer MJ and Fuchs CS. Physical activity, obesity, height, and the risk of pancreatic cancer. JAMA. 2001;286: 921–929. 11509056 10.1001/jama.286.8.921

[6] Whitcomb DC, Gorry MC, Preston RA, Furey W, Sossenheimer MJ, Ulrich CD, et al Hereditary pancreatitis is caused by a mutation in the cationic trypsinogen gene. Nat Genet. 1996;14: 141–145. 8841182 10.1038/ng1096-141

[7] Jaffee EM, Hruban RH, Canto M and Kern SE. Focus on pancreas cancer. Cancer Cell. 2002;2: 25–28. 12150822 10.1016/s1535-6108(02)00093-4

[8] Lohr M, Maisonneuve P and Lowenfels AB. K-Ras mutations and benign pancreatic disease. Int J Pancreatol. 2000;27: 93–103. 10862508 10.1385/IJGC:27:2:093

[9] Farrow B, Albo D and Berger DH. The role of the tumor microenvironment in the progression of pancreatic cancer. J Surg Res. 2008;149: 319–328. 10.1016/j.jss.2007.12.757 18639248

[10] Lindsay TH, Jonas BM, Sevcik MA, Kubota K, Halvorson KG, Ghilardi JR, et al Pancreatic cancer pain and its correlation with changes in tumor vasculature, macrophage infiltration, neuronal innervation, body weight and disease progression. Pain. 2005;119: 233–246. 16298491 10.1016/j.pain.2005.10.019

[11] Lee RC, Feinbaum RL and Ambros V. The C. elegans heterochronic gene lin-4 encodes small RNAs with antisense complementarity to lin-14. Cell. 1993;75: 843–854. 8252621 10.1016/0092-8674(93)90529-y

[12] Zhao L, Chen X and Cao Y. New role of microRNA: carcinogenesis and clinical application in cancer. Acta Biochim Biophys Sin (Shanghai). 2011;43: 831–839. 10.1093/abbs/gmr080 21908856

[13] Hagan JP and Croce CM. MicroRNAs in carcinogenesis. Cytogenet Genome Res. 2007;118: 252–259. 18000378 10.1159/000108308

[14] Liao WT, Li TT, Wang ZG, Wang SY, He MR, Ye YP, et al microRNA-224 promotes cell proliferation and tumor growth in human colorectal cancer by repressing PHLPP1 and PHLPP2. Clin Cancer Res. 2013;19: 4662–4672. 10.1158/1078-0432.CCR-13-0244 23846336

[15] Jung HM, Patel RS, Phillips BL, Wang H, Cohen DM, Reinhold WC, et al Tumor suppressor miR-375 regulates MYC expression via repression of CIP2A coding sequence through multiple miRNA-mRNA interactions. Mol Biol Cell. 2013;24: 1638–1648, S1631–1637. 10.1091/mbc.E12-12-0891 23552692 PMC3667718

[16] Han Z, Yang Q, Liu B, Wu J, Li Y, Yang C, et al MicroRNA-622 functions as a tumor suppressor by targeting K-Ras and enhancing the anticarcinogenic effect of resveratrol. Carcinogenesis. 2012;33: 131–139. 10.1093/carcin/bgr226 22016468

[17] Zhao G, Zhang JG, Liu Y, Qin Q, Wang B, Tian K, et al miR-148b functions as a tumor suppressor in pancreatic cancer by targeting AMPKalpha1. Mol Cancer Ther. 2013;12: 83–93. 10.1158/1535-7163.MCT-12-0534-T 23171948

[18] El Tayebi HM, Hosny KA, Esmat G, Breuhahn K and Abdelaziz AI. miR-615-5p is restrictedly expressed in cirrhotic and cancerous liver tissues and its overexpression alleviates the tumorigenic effects in hepatocellular carcinoma. FEBS Lett. 2012;586: 3309–3316. 10.1016/j.febslet.2012.06.054 22819824

[19] Altomare DA, Tanno S, De Rienzo A, Klein-Szanto AJ, Tanno S, Skele KL, et al Frequent activation of AKT2 kinase in human pancreatic carcinomas. J Cell Biochem. 2002;87: 470–476. 14735903 10.1002/jcb.10287

[20] Ruggeri BA, Huang L, Wood M, Cheng JQ and Testa JR. Amplification and overexpression of the AKT2 oncogene in a subset of human pancreatic ductal adenocarcinomas. Mol Carcinog. 1998;21: 81–86. 9496907

[21] Cheng JQ, Ruggeri B, Klein WM, Sonoda G, Altomare DA, Watson DK, et al Amplification of AKT2 in human pancreatic cells and inhibition of AKT2 expression and tumorigenicity by antisense RNA. Proc Natl Acad Sci U S A. 1996;93: 3636–3641. 8622988 10.1073/pnas.93.8.3636 PMC39663

[22] Asano T, Yao Y, Zhu J, Li D, Abbruzzese JL and Reddy SA. The PI 3-kinase/Akt signaling pathway is activated due to aberrant Pten expression and targets transcription factors NF-kappaB and c-Myc in pancreatic cancer cells. Oncogene. 2004;23: 8571–8580. 15467756 10.1038/sj.onc.1207902

[23] Yamamoto S, Tomita Y, Hoshida Y, Morooka T, Nagano H, Dono K, et al Prognostic significance of activated Akt expression in pancreatic ductal adenocarcinoma. Clin Cancer Res. 2004;10: 2846–2850. 15102693 10.1158/1078-0432.ccr-02-1441

[24] Koorstra JB, Hustinx SR, Offerhaus GJ and Maitra A. Pancreatic carcinogenesis. Pancreatology. 2008;8: 110–125. 10.1159/000123838 18382097 PMC2663569

[25] Zhao WG, Yu SN, Lu ZH, Ma YH, Gu YM and Chen J. The miR-217 microRNA functions as a potential tumor suppressor in pancreatic ductal adenocarcinoma by targeting KRAS. Carcinogenesis. 2010;31: 1726–1733. 10.1093/carcin/bgq160 20675343

[26] Yamamichi N, Shimomura R, Inada K, Sakurai K, Haraguchi T, Ozaki Y, et al Locked nucleic acid in situ hybridization analysis of miR-21 expression during colorectal cancer development. Clin Cancer Res. 2009;15: 4009–4016. 10.1158/1078-0432.CCR-08-3257 19509156

[27] Chehrehasa F, Meedeniya AC, Dwyer P, Abrahamsen G and Mackay-Sim A. EdU, a new thymidine analogue for labelling proliferating cells in the nervous system. J Neurosci Methods. 2009;177: 122–130. 10.1016/j.jneumeth.2008.10.006 18996411

[28] Yu S, Lu Z, Liu C, Meng Y, Ma Y, Zhao W, et al miRNA-96 suppresses KRAS and functions as a tumor suppressor gene in pancreatic cancer. Cancer Res. 2010;70: 6015–6025. 10.1158/0008-5472.CAN-09-4531 20610624

[29] Li J, Wan Y, Guo Q, Zou L, Zhang J, Fang Y, et al Altered microRNA expression profile with miR-146a upregulation in CD4+ T cells from patients with rheumatoid arthritis. Arthritis Res Ther. 2010;12: R81 10.1186/ar3006 20459811 PMC2911863

[30] Lois C, Hong EJ, Pease S, Brown EJ and Baltimore D. Germline transmission and tissue-specific expression of transgenes delivered by lentiviral vectors. Science. 2002;295: 868–872. 11786607 10.1126/science.1067081

[31] Zhu S, Wu H, Wu F, Nie D, Sheng S and Mo YY. MicroRNA-21 targets tumor suppressor genes in invasion and metastasis. Cell Res. 2008;18: 350–359. 10.1038/cr.2008.24 18270520

[32] Ma Y, Yu S, Zhao W, Lu Z and Chen J. miR-27a regulates the growth, colony formation and migration of pancreatic cancer cells by targeting Sprouty2. Cancer Lett. 2010;298: 150–158. 10.1016/j.canlet.2010.06.012 20638779

[33] Verheijen R, Kuijpers HJ, Schlingemann RO, Boehmer AL, van Driel R, Brakenhoff GJ, et al Ki-67 detects a nuclear matrix-associated proliferation-related antigen. I. Intracellular localization during interphase. J Cell Sci. 1989;92 (Pt 1): 123–130. 2674163 10.1242/jcs.92.1.123

[34] Xiao F, Zuo Z, Cai G, Kang S, Gao X and Li T. miRecords: an integrated resource for microRNA-target interactions. Nucleic Acids Res. 2009;37: D105–110. 10.1093/nar/gkn851 18996891 PMC2686554

[35] Lu J, Getz G, Miska EA, Alvarez-Saavedra E, Lamb J, Peck D, et al MicroRNA expression profiles classify human cancers. Nature. 2005;435: 834–838. 15944708 10.1038/nature03702

[36] Jay C, Nemunaitis J, Chen P, Fulgham P and Tong AW. miRNA profiling for diagnosis and prognosis of human cancer. DNA Cell Biol. 2007;26: 293–300. 17504025 10.1089/dna.2006.0554

[37] Hulf T, Sibbritt T, Wiklund ED, Bert S, Strbenac D, Statham AL, et al Discovery pipeline for epigenetically deregulated miRNAs in cancer: integration of primary miRNA transcription. BMC Genomics. 2011;12: 54 10.1186/1471-2164-12-54 21255435 PMC3037319

[38] Jamieson NB, Morran DC, Morton JP, Ali A, Dickson EJ, Carter CR, et al MicroRNA molecular profiles associated with diagnosis, clinicopathologic criteria, and overall survival in patients with resectable pancreatic ductal adenocarcinoma. Clin Cancer Res. 2012;18: 534–545. 10.1158/1078-0432.CCR-11-0679 22114136

[39] Nelson PT and Wilfred BR. In situ hybridization is a necessary experimental complement to microRNA (miRNA) expression profiling in the human brain. Neurosci Lett. 2009;466: 69–72. 10.1016/j.neulet.2009.04.044 19393719 PMC3171000

[40] Sempere LF, Preis M, Yezefski T, Ouyang H, Suriawinata AA, Silahtaroglu A, et al Fluorescence-based codetection with protein markers reveals distinct cellular compartments for altered MicroRNA expression in solid tumors. Clin Cancer Res. 2010;16: 4246–4255. 10.1158/1078-0432.CCR-10-1152 20682703 PMC3229296

[41] Gray S, Pandha HS, Michael A, Middleton G and Morgan R. HOX genes in pancreatic development and cancer. JOP. 2011;12: 216–219. 21546695

[42] Coolen MW, Stirzaker C, Song JZ, Statham AL, Kassir Z, Moreno CS, et al Consolidation of the cancer genome into domains of repressive chromatin by long-range epigenetic silencing (LRES) reduces transcriptional plasticity. Nat Cell Biol. 2010;12: 235–246. 10.1038/ncb2023 20173741 PMC3058354

[43] Gao W, Gu Y, Li Z, Cai H, Peng Q, Tu M, et al miR-615-5p is epigenetically inactivated and functions as a tumor suppressor in pancreatic ductal adenocarcinoma. Oncogene. 2014;0. 10.1038/onc.2014.101 24769899

[44] Lewis BP, Burge CB and Bartel DP. Conserved seed pairing, often flanked by adenosines, indicates that thousands of human genes are microRNA targets. Cell. 2005;120: 15–20. 15652477 10.1016/j.cell.2004.12.035

[45] Bond-Smith G, Banga N, Hammond TM and Imber CJ. Pancreatic adenocarcinoma. BMJ. 2012;344: e2476 10.1136/bmj.e2476 22592847

[46] Wolfgang CL, Herman JM, Laheru DA, Klein AP, Erdek MA, Fishman EK, et al Recent progress in pancreatic cancer. CA Cancer J Clin. 2013;63: 318–348. 10.3322/caac.21190 23856911 PMC3769458

[47] Gilad S, Meiri E, Yogev Y, Benjamin S, Lebanony D, Yerushalmi N, et al Serum microRNAs are promising novel biomarkers. PLoS One. 2008;3: e3148 10.1371/journal.pone.0003148 18773077 PMC2519789

[48] Bloomston M, Frankel WL, Petrocca F, Volinia S, Alder H, Hagan JP, et al MicroRNA expression patterns to differentiate pancreatic adenocarcinoma from normal pancreas and chronic pancreatitis. JAMA. 2007;297: 1901–1908. 17473300 10.1001/jama.297.17.1901

[49] Camps C, Buffa FM, Colella S, Moore J, Sotiriou C, Sheldon H, et al hsa-miR-210 Is induced by hypoxia and is an independent prognostic factor in breast cancer. Clin Cancer Res. 2008;14: 1340–1348. 10.1158/1078-0432.CCR-07-1755 18316553

