# Correction: MiRNA-615-5p Functions as a Tumor Suppressor in Pancreatic Ductal Adenocarcinoma by Targeting AKT2

**DOI:** 10.1371/journal.pone.0128257

**Published:** 2015-05-08

**Authors:** Yang Sun, Tingting Zhang, Cuiping Wang, Xianglan Jin, Congwei Jia, Shuangni Yu, Jie Chen

There is an error in Table 2. The values for “Negative,” “Weak positive,” and “Positive rate” in the “PDAC” column are incorrect. Please see the corrected Table 2 here.

**Table 2:** miR-615-5p expression in PDAC and normal pancreas tissues determined by locked nucleic acid *in situ* hybridization (LNA-ISH)

miR-615-5p expression	PDAC(n = 97)	Normal pancreas(n = 97)
Negative	42	7
Weak positive	32	40
Strong positive	23	50
Positive rate	56.7%(55/97)	92.8%(90/97)**

***Note*:** **P <0.01 PDAC tissue *vs*. normal pancreas tissues (Fisher’s exact test). PDAC: Pancreatic ductal adenocarcinoma.
