# Supplementary material for: MiRNA-615-5p Functions as a Tumor Suppressor in Pancreatic Ductal Adenocarcinoma by Targeting AKT2
Source: PLoS One. 2015 Apr 9;10(4):e0119783. doi: 10.1371/journal.pone.0119783 (PMC4391776; doi:10.1371/journal.pone.0119783)
Supplement: S1 Checklist — (DOC) [file pone.0119783.s001.doc]

**ARRIVE:** Animal research: Reporting *in vivo* experiments

| **TITLE** | 1 | miR-615 overexpression inhibits tumor growth and metastasis in *vivo* |
| --- | --- | --- |
| **ABSTRACT** | 2 | BACKGROUND AND OBJECTIVE: *In vitro*, miR-615-5p can inhibit proliferation of two pancreatic cancer cell lines (PANC-1 and MIA PaCa-2 cells). But its effects *in vivo* are undetermined yet. The purpose of this study was to evaluate the effect of miR-615 on tumor growth and metastatic potential in *vivo*.  METHODS: MIA PaCa-2 cells stably overexpressing miR-615 and the mock vector were established. Subcutaneously injection into the dorsal flanks of BALB/c nude mice was used to generate tumor xenograft models. Tail vein injection was used to generate contrived metastatic models.  RESULTS: 7 weeks after inoculation, tumor volume in the LV-miR-615-MIA group was lower compared with control animals (393.10±132.20 mm^3^ *vs.* 738.04±222.12 mm^3^, *P*<0.01) and tumor weight (0.79±0.26 g *vs.* 1.48±0.44 g, *P*<0.01) were significantly decreased in mice that received miR-615- overexpressing cells compared with the controls. Mice injected with LV-miR-615-MIA cells showed reduced number of tumor nodules in the liver compared with animals injected with control cells (3.8±2.8 *vs.* 15.5±5.4, *P*<0.01).  CONCLUSIONS: miR-615 overexpression may inhibit pancreatic cancer growth by blocking proliferation and promoting apoptosis, indicating its role in suppressing tumor metastasis. |
| **INTRODUCTION** | | |
| **Background** | 3 | a. Pancreatic ductal adenocarcinoma (PDAC) is a highly invasive cancer with a poor prognosis. In the present study, miR-615-5p expression was significantly lower in human PDAC than in adjacent normal tissues. AKT2 expression is inversely correlated with miR-615-5p in PDAC tissues. Furthermore, miR-615-5p may function as a tumor suppressor in pancreatic cancer cells *in vitro*. |
|  |  | b. Nude mice lack intact immune system, which can play a prohibitive role in tumor development.  Subcutaneously injection into the dorsal flanks of BALB/c nude mice was used to generate tumor xenograft models. Tail vein injection was used to generate contrived metastatic models. |
| **Objectives** | 4 | miR-615-5p plays a crucial role in numerous biological functions, and is linked to pancreatic cancer cell proliferation and invasion and migration in *vitro*. However, the effects of stable overexpression of miR-615 on tumor growth and metastasis i*n vivo* are poorly understood. Here we hypothesed that miR-615 can inhibit the capability of pancreatic cancer development and metastasis *in vivo*. The purpose of this study was to evaluate the effect of miR-615 on tumor growth and metastatic potential in *vivo*. |
| **METHODS** | | |
| **Ethical statement** | 5 | All procedures and animal experiments were approved by the Animal Care and Use Committee of Peking Union Medical College (Approval No. XHDW-2012-016) and conducted in accordance with all state regulations. |
|  |  | a. **Tumor xenograft models:**  LV-miR-615-MIA or LV-control-MIA cells (6 × 10^6^ cells in 200ul PBS per mouse) were subcutaneously inoculated into the dorsal flanks of mice (n=10). Mice not successfully modeled or died were excluded. n=8 in LV-control-MIA group; n=6 in LV-miR-615-MIA group  **Metastatic models:**  LV-miR-615-MIA or LV-control-MIA cells (1 × 10^5^ cells resuspended in 200 µL PBS per mouse) were injected by tail-vein to nude mice (6 LV-control-MIA and 6 stably overexpressing mir-615 MIA cell lines) |
|  |  | b. Mice were randomly divided into experimental and control groups using random number table to minimize the effects of subjective bias when allocating animals to treatment.  **Tumor xenograft models:** Two independent reviewers were blinded to the grouping when measuring tumor volume, tumor weight, the numbers of apoptotic cells and the percentages of Ki-67-positive cells.  **Metastatic models:** Two independent pathologists were blinded to the grouping when counting tumor nodules in the liver tissues. |
|  |  | c. The experimental unit is a single animal.  **Tumor xenograft models** (MIA PaCa-2 cells transduced with lentivirus pLV-miR-615 (LV-miR-615-MIA) or pLV-miR-mock (LV-control-MIA) (6 × 10^6^ cells) were subcutaneously inoculated into the dorsal flanks of BALB/c nude mice.):  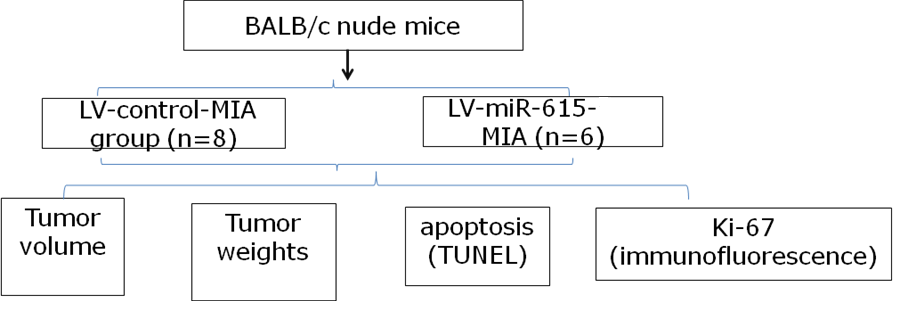  **Metastatic models** (LV-miR-615-MIA or LV-control-MIA cells were injected into nude mice via tail vein.):  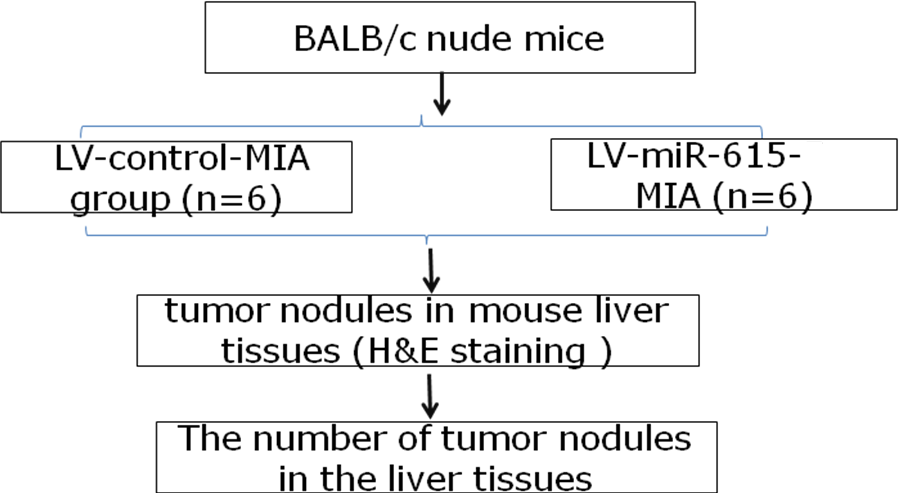 |
| **Experimental procedures** | 7 | For each experiment and each experimental group, including controls, provide precise details of all procedures carried out.  MiR-615-overexpressing lentiviral constructs were generated using synthetic oligonucleotides. Every mouse was weighed. LV-miR-615-MIA or LV-control-MIA cells (6 × 10^6^ cells in 200ul PBS per mouse) were subcutaneously inoculated into the dorsal flanks of mice (10 LV-control-MIA cells and 10 stably overexpressing mir-615-MIA cells).  LV-miR-615-MIA or LV-control-MIA cells (1 × 10^5^ cells resuspended in 200 µL PBS per mouse) were injected by tail-vein to nude mice (6 LV-control-MIA and 6 stably overexpressing mir-615 MIA cell lines) after intraperitoneal injection of 3% pentobarbital sodium (2 ml/kg body weight) for anesthesia . |
|  |  | b.  In the subcutaneous tumor xenograft models, body weight and tumor volume were monitored every 3 days. The animals were killed 49 days after inoculation of tumor cells. All tumor grafts were excised, weighed, harvested, fixed and embedded.  In the metastatic models, body weight was monitored every 3 days. 4 weeks after tail injection, animals were killed, and livers were harvested to count tumor nodules and evaluate tumor metastasis. |
|  |  | c. Operating in the laboratory |
|  |  | d. LV-miR-615-MIA or LV-control-MIA cells (6 × 10^6^ cells in 200ul PBS per mouse) were **subcutaneously inoculated into the dorsal flanks of mice** (10 LV-control-MIA cells and 10 stably overexpressing mir-615-MIA cells) **[25]**.  LV-miR-615-MIA or LV-control-MIA cells (1 × 10^5^ cells resuspended in 200 µL PBS per mouse) were **injected by tail-vein** to nude mice (6 LV-control-MIA and 6 stably overexpressing mir-615 MIA cell lines)  Subcutaneous injection did not use anesthesia, and tail intravenous injection after intraperitoneal injection of 3% pentobarbital sodium (2 ml/kg body weight) for anesthesia. |
| **Experimental animals** | 8 | a. Six-week-old male BALB/c nude male mice , body weight: 20.34±2.19 (17.1～25.3) |
|  |  | b.  purchased from Vital River Laboratory Animal Technology Co., Ltd., (Beijing, China)  Designation:  CanN.Cg-Foxn1nu/CrlVr，the Charles River Japan（CRJ）company obtained the strain through Mating and backcross of BALB∕cABon-nu and BALB∕cAnNCrj-nu。This was inbreed strain; genetic monitoring confirmed is BALB/c nude mice. The mice didn’t have thymus, therefore had T cell immune defects. |
| **Housing and husbandry** | 9 | a. Housing was specific pathogen free (SPF). Type of cage：Plastic cage with sealed air filtration device, including a standard transparent plastic cage, with overlying air filtration material. Bedding material：Chinese Academy of Sciences (CAS) SLAC into sterile bag litter, The need for surface disinfection (0.1% peracetic acid) when used； number of cage companions：4 mice/cage ；tank shape and material：Square transparent high voltage box, bottom area 97 square centimeters and a height of 12.7 cm. |
|  |  | b.  light/dark cycle：Artificial lighting，Light-dark cycle-10 hours of light and 14 hours of dark every day； temperature：at room temperature for 26 ~ 28 ℃ (78 ~ 82ºF)；Relative humidity of 40 to 60%；Per hour with a high efficiency filter (99.9%) of 10 to 15 times ventilation； quality of water：Sterile water；type of food：CAS SLAC adult rat feed cobalt 60 sterile bags packed；access to food and water：Free feeding and drinking；environmental enrichment：Nothing to do with this experiment |
|  |  | c.  Evaluation of the benefits for the experimental animals: (1) The experiment, in which the needs of the animals were fully considered, was approved by the ethics committee, including physiological (adequate food, water, temperature and illumination), environmental, psychological and social needs (socially raised, 4-6 animals per cage, avoid tiredness and overstimulation). The outcomes of the preliminary experiment and the literatures were taken into consideration to make rational design of the sample size and operation standard. (2) A daily observation was preformed to prevent the animals from anger, comfortlessness, fear, nervousness, pain or damage and to keep them at normal status. Abuse, excessive or incorrect medication was avoided. For subcutaneous injection, which was easy to operate, narcotics were not applicable; for tail vein injection, which was not easy to operate, intraperitoneal anesthesia was given to alleviate the pain of the animals. (3) At the time of endpoint, the animals were slaughtered within 15 s to avoid the nervousness of the other animals. |
| **Sample size** | 10 | a. 20 nude mice were used to examine tumorigenicity (10 for treatment group, 10 for control) and 12 nude mice were used to examine metastasis (6 for treatment group, 6 for control). |
|  |  | b. the number of animals in each group was mainly based on the pre-experiment results, experimental period, and statistical requirements.  Statistical requirements at least six mice in each group.  Observation time of tail vein injection was short, so in accordance with the statistical requirements, minimum amount 6 mice / group.  Preliminary experiments found that not all mice with subcutaneous injection formed tumors at about two weeks after injection; a longer observation time of seven weeks was adopted, therefore 10 mice /group were used in this experiment to ensure that the statistically required number of animals at the end of the experiment. |
|  |  | c. three independent replications  . |
| **Allocating animals to experimental groups** | 11 | a. The mice were randomly divided into two groups using random number table. |
|  |  | b.   **Subcutaneous tumor xenograft models**:  LV-miR-615-MIA or LV-control-MIA cells (6 × 10^6^ cells in 200ul PBS per mouse) were subcutaneously inoculated into the dorsal flanks of mice (10 LV-control-MIA cells and 10 stably overexpressing mir-615-MIA cells).  **Metastatic models:**:  LV-miR-615-MIA or LV-control-MIA cells (1 × 10^5^ cells resuspended in 200 µL PBS per mouse) were injected by tail-vein to nude mice (6 LV-control-MIA and 6 stably overexpressing mir-615 MIA cell lines) |
| **Experimental outcomes** | 12 | 1. Tumor dimensions were measured by calipers every 3 days, and the volumes estimated using the formula for hemi-ellipsoids: V = length (mm) × width (mm) × height (mm) × 0.5236 . For end-point experiments, tumors were removed and weighed 6 weeks after tumor cell injection. 2. Four weeks after injection, animals were euthanized by cervical dislocation, and livers were harvested to count tumor nodules and evaluate tumor metastasis (H&E staining). 3. Indirect immunofluorescence staining of Ki67 was used on mouse tumor tissue sections . Deparaffinization, endogenous peroxidase inactivation, antigen retrieval of FFPE clinical tissues, and immunostaining with mouse monoclonal antibodies (sc-23900, Santa Cruz Biotech, USA) were performed. Goat anti-mouse IgG secondary antibodies conjugated to rhodamine (115-025-003, Jackson, USA) were used for detection. 4. Apoptosis in mouse tumor tissues was measured using the In Situ Cell Death Detection Kit (1684809, Roche, CHE), according to the supplier’s instructions. |
| **Statistical methods** | 13 | a.  Statistical analyses were performed using the SPSS statistical software 17.0 (SPSS Inc., Chicago, IL, USA). Normally distributed data are presented as mean ± standard deviation (SD). Statistical significance was evaluated by Student's *t* test. |
|  |  | b. single animal. |
|  |  | c.  Normally distributed data are presented as mean ± standard deviation (SD). |
| **RESULTS** | | |
| **Baseline data** | 14 | **Subcutaneous tumor xenograft models**:  Body weight before treatment: LV-miR-615-MIA group: 21.33±2.38 g，LV-control-MIA group： 21.71±2.23 g；  **Metastatic models:**:  Body weight before treatment: LV-miR-615-MIA group: 19.16±0.47 g；LV-control-MIA group： 18.53±1.76 g； |
| **Numbers analysed** | 15 | a.  **Subcutaneous tumor xenograft models:** n=8 (8/10) in LV-control-MIA group; n=6 (6/10) in LV-miR-615-MIA group.  **Metastatic models:** n=6 (6/6) in LV-control-MIA group; n=6 (6/6) in LV-miR-615-MIA group |
|  |  | b. Mice were excluded due to nature die or not tumor formation.. |
| **Outcomes and estimation** | 16 | **Outcomes:** p335-358 in the manuscript.  **Subcutaneous tumor xenograft models:** The data are shown as mean±standard deviation (SD) (n=8 in LV-control-MIA group; n=6 in LV-miR-615-MIA group).  **Metastatic models:** The data are shown as mean±SD (n=6 in LV-control-MIA group; n=6 in LV-miR-615-MIA group) |
| **Adverse events** | 17 | a. NO |
|  |  | b.  NO |
| **DISCUSSION** | | |
| **Interpretation/scientific implications** | 18 | a. P466-475 in the manuscript. |
|  |  | b. p475-480 in the manuscript. |
|  |  | c. miR-615 attenuates the malignant behavior of PDAC cells, thus, it may be a promising therapeutic target in the future (p474-475 in the manuscript). |
| **Generalisability/ translation** | 19 | miR-615 attenuates the malignant behavior of PDAC cells, thus, it may be a promising therapeutic target in the future (p474-475 in the manuscript). |
| **Funding** | 20 | This study was supported by the National Nature Science Foundation of China (grant 30973470, 81172334), the National Science and Technology Support Project (the 11th Five-Year Plan) of China (grant 2006BAI02A14), and the Scientific Research Special Projects of Health Industry of China (grant 200802011). |

***Note:***

**Reference**

Carol Kilkenny, William Browne, Innes C Cuthill, et al. Animal research: Reporting *in vivo* experiments: The ARRIVE guidelines. British Journal of Pharmacology 2010,160:1577–1579.
