# Supplementary material for: MiRNA-615-5p Functions as a Tumor Suppressor in Pancreatic Ductal Adenocarcinoma by Targeting AKT2
Source: PLoS One. 2015 Apr 9;10(4):e0119783. doi: 10.1371/journal.pone.0119783 (PMC4391776; doi:10.1371/journal.pone.0119783)
Supplement: S1 File — Contains, Table A: Primers and oligonucleotides used for the Dual Luciferase Reporter Assay. Table B: Correlation between miR-615-5p expression and multiple clinicopathological characteristics, including AKT2 expression, in 72 follow-up PDAC patients. (DOCX) [file pone.0119783.s002.docx]

**Table A.** Primers and oligonucleotides used for the Dual Luciferase Reporter Assay

| AKT2 WT F | 5’-AGCTCGCTAGCCTCGAGCTGCTCTTTTTGTGTAATCTTTTCCAGTTAATG-3’ |
| --- | --- |
| AKT2 WT R | 5’-TGCAGGTCGACTCTAGATTTTACAGATGGATAGCTAGTTTATTACAGGAC-3’ |
| AKT2 MUT F | 5’-CCATGGGGTAAGTTCGTCAACCGCTTCAGCTGCCCCGTTAAC-3’ |
| AKT2 MUT R | 5’-CAGCTGAAGCGGTTGACGAACTTACCCCATGGAACCCCAGGAAG-3’ |
| miR-615-5p sense | 5’-TCGAGGATCCGAGCACCGGGGACCCCCT-3’ |
| miR-615-5p antisense | 5’-CTAGAGGGGGTCCCCGGTGCTCGGATCC-3’ |

**Table B** Correlation between miR-615-5p expression and multiple clinicopathological characteristics, including AKT2 expression, in 72 follow-up PDAC patients

| Characteristics | N | miR-615-5p, N(%) | | *P* |
| --- | --- | --- | --- | --- |
|  |  | Negative | Positive |  |
| Sex |  |  |  |  |
| Male | 45 | 16（35.6） | 29（64.4） | 0.660 |
| Female | 27 | 11（40.7） | 16（59.3） |  |
| Age |  |  |  |  |
| <60 | 32 | 13（40.6） | 19（59.4） | 0.624 |
| ≥60 | 40 | 14（35.0） | 26（65.0） |  |
| Location |  |  |  |  |
| head | 52 | 18（34.6） | 34（65.4） | 0.415 |
| body/tail | 20 | 9（45.0） | 11（55.0） |  |
| Pancreaticobiliary ductal infiltration |  |  |  |  |
| Yes | 35 | 18（51.4） | 17（48.6） | 0.018 |
| No | 37 | 9（24.3） | 28（75.7） |  |
| Peritoneal metastasis |  |  |  |  |
| Yes | 4 | 2（50.0） | 2（50.0） | 0.595 |
| No | 68 | 25（36.8） | 43（63.2） |  |
| T classification |  |  |  |  |
| T1 | 4 | 3（75.0） | 1（25.0） | 0.075 |
| T2 | 9 | 3（33.3） | 6（66.7） |  |
| T3 | 52 | 16（30.8） | 36（69.2） |  |
| T4 | 7 | 5（71.4） | 2（28.6） |  |
| Differentiation state |  |  |  |  |
| High | 15 | 1（6.7） | 14（93.3） | 0.021 |
| Medium | 37 | 17（45.9） | 20（54.1） |  |
| Low | 20 | 9（45.0） | 11（55.0） |  |
| Clinical stage classification |  |  |  |  |
| I/IIA | 30 | 8（26.7） | 22（73.3） | 0.109 |
| IIB/III/IV | 42 | 19（45.2） | 23（54.8） |  |
| Node metastasis |  |  |  |  |
| N0 | 34 | 11（32.4） | 23（67.6） | 0.393 |
| N1 | 38 | 16（42.1） | 22（57.9） |  |
| AKT2 expression |  |  |  |  |
| Negative | 34 | 8（23.5） | 26（76.4） | 0.015(Kendall correlation analysis) |
| Positive | 38 | 19（50.0） | 19（50.0） |  |
